# Political Ideologies, Government Trust, and COVID-19 Vaccine Hesitancy in South Korea: A Cross-Sectional Survey

**DOI:** 10.3390/ijerph182010655

**Published:** 2021-10-12

**Authors:** Hyun Kyung Park, Ji Hye Ham, Deok Hyun Jang, Jin Yong Lee, Won Mo Jang

**Affiliations:** 1 Health Insurance Research Institute, National Health Insurance Service, Wonju 26464, Korea; hpark@nhis.or.kr; 2 Department of Urban Health and Policy, Seoul Health Foundation, Seoul 04512, Korea; redmoon@seoulhealth.kr; 3 Research Analytics & Communications, Gallup Korea, Seoul 03167, Korea; dhjang@gallup.co.kr; 4 Public Healthcare Center, Seoul National University Hospital, Seoul 03080, Korea; jylee2000@gmail.com; 5 Department of Health Policy and Management, Seoul National University College of Medicine, Seoul 03080, Korea; 6 Department of Public Health and Community Medicine, Seoul Metropolitan Government-Seoul National University Boramae Medical Centre, Seoul 07061, Korea

**Keywords:** vaccine hesitancy, COVID-19 vaccine, political ideology, trust

## Abstract

This study aimed to assess the correlation between political ideologies, government trust, and COVID-19 vaccine hesitancy in South Korea during the COVID-19 pandemic. A cross-sectional survey was conducted among South Korea’s general population and 1000 respondents (aged 18 years and older) were included. We used multivariate logistic regression models to identify the factors associated with vaccine hesitancy. Respondents who self-identified as liberal or held “no political opinion” had higher rates of vaccine hesitancy than conservative respondents. People’s trust in the government’s countermeasures was associated with vaccination. Respondents who had risk perceptions (affective and cognitive) of COVID-19 had lower rates of vaccine hesitancy. Perceptions that the vaccine was not safe and being aged 18–29, 30–39, or 40–49 were associated with a higher probability of vaccine hesitancy. This study found that even if vaccine safety and risk perceptions toward COVID-19 were adjusted, self-rated political ideologies and government trust was associated with COVID-19 vaccine hesitancy. More effort to communicate with those who are *liberal or “no political opinion”, younger, and have lower level of trust in the government are required to dissolve vaccine hesitancy. Further studies should analyze the mechanism of COVID-19 vaccine uptake for effective herd immunity.

## 1. Introduction

After the first case of COVID-19 was confirmed by the World Health Organization (WHO) in China at the end of 2019, the number of confirmed cases of COVID-19 in South Korea exceeded 223,000 by August 2021 [1]. On 30 January 2020, the WHO declared that the COVID-19 outbreak was a Public Health Emergency of International Concern (PHEIC). By March 11, they declared the outbreak to be a pandemic. Since early 2020, the rush to create effective COVID-19 vaccines and distribute them globally has highlighted the need for effective dissemination of public health information. The South Korean government unveiled its vaccination plan at the end of January 2021 [2]. By the end of August 2021, more than 11.4 million people had been fully vaccinated against COVID-19 in South Korea [1]. Vaccinations are widely regarded as one of the most essential and successful public health measures to control and prevent diseases [3,4]. However, in South Korea, the public’s perceptions of the safety of the COVID-19 vaccine (e.g., receiving a mutated version of the coronavirus, suffering from serious side effects, etc.) and the government’s credibility led to tremendously low inoculation rates; only 5% of South Koreans were vaccinated by 28 February 2021 [5,6]. 

Inoculation rates are associated with vaccine hesitancy or people’s hesitancy to be inoculated [7]. Vaccine hesitancy ranked among the WHO’s top 10 threats to global health in 2019 [8]. Vaccine hesitancy is affected by factors such as individuals’ confidence, complacency, and convenience. This is referred to as the “three Cs model” [3,9,10,11,12]. Some studies have shown that vaccine hesitancy is affected by political factors as well [11,13,14,15]. In general, these studies assert that the more conservative people’s political views and tendencies are, the more reserved and progressive the propensity for vaccination, and the more active the attitude of vaccination. Furthermore, there are reports that people’s trust in governments and expert groups also affects their vaccine hesitancy [16,17,18]. Specifically, the lower someone’s trust in the government or expert groups, the higher their vaccine hesitancy. 

There are grounds for this kind of study in a South Korean context. In particular, many South Koreans refused to get their normal influenza vaccines in 2020 due to their political views [19]. As a result, many South Korean experts are raising concerns about South Koreans’ vaccine hesitancy as the COVID-19 vaccines are developed and distributed. In short, there are few studies that explicitly correlate COVID-19 vaccine hesitancy and people’s self-described political beliefs and/or their lack of trust in the government. Furthermore, studies on people’s risk perception regarding diseases (complacency regarding the three Cs) and their perceptions of a vaccine’s safety (confidence regarding the three Cs) lack detail and need to be revised [13,16,17,18,19,20,21]. Studies have shown that the influence of the president’s remarks, which are supported by individual political tendencies, can also affect vaccine hesitancy. For example, when the U.S. President issued a vaccination statement, his opponents hesitated to get vaccinated) [13,14,22].

Therefore, this study analyzes the correlation between people’s hesitancy to receive a hypothetical COVID-19 vaccine, their self-described political views, and their trust in the government. Based on the previous findings, we aim to verify the following hypotheses: (1) conservative political ideology would be related to high vaccine hesitancy and (2) low levels of government trust would be associated with high vaccine hesitancy.

## 2. Materials and Methods

### 2.1. Participants

We analyzed data from a single survey conducted between February 16 and 18, 2021. A nationwide sample of 1000 individuals aged 18 or older were selected using post-stratification according to gender, age, and region. Of the 20,054 eligible cases, 6841 were contacted, of whom 1000 responded. Trained interviewers conducted all interviews via computer-assisted telephone interviews (CATI; 85% on mobile phones, and 15% on landlines) using random digit dialing numbers (RDD). Weight was calculated to represent the general population. The total number of weighted cases in this study equaled the total number of unweighted cases at the national level. The survey was conducted by Gallup Korea, an affiliate of Gallup International. More detailed information on the survey is available in Table S1 of Supplementary File S1.

The survey also recorded respondents’ demographic characteristics, including their gender, age, occupation, self-reported household economic status, region, and political ideology. Age was classified into five levels (aged 18–29, 30–39, 40–49, 50–59, and 60+ years). Occupation was classified into three levels (unemployed, employed, and full-time homemakers or students). Self-reported household economic status was classified into three levels (upper, middle, and lower). Respondents were classified into five regions (Yeongnam, a south-eastern region that has supported the current major opposition party since 1988; Honam, a southwestern region that has supported the current governing party since 1988; Capital Metro, a Seoul metropolitan area [Seoul, Incheon, and Gyeonggi Province]; Chungcheong; and Gangwon/Jeju), according to the regional voting model, a concept often used to explain why voters support local parties in South Korea [23,24]. Respondents’ self-described political ideologies were classified into four levels: conservative, liberal, moderate, and no political opinion.

### 2.2. Survey Instruments

Vaccine hesitancy, the outcome measure, was assessed using the following question: “If COVID-19 vaccines are introduced in Korea, will you receive or not receive the COVID-19 vaccine?” The responses of “I will definitely receive the vaccine” or “I will probably receive the vaccine” were classified as “Will receive the vaccine,” and the responses of “I will probably not receive the vaccine” or “I will definitely not receive the vaccine” were classified as “Will not receive the vaccine.”

The independent variables in this study included gender, age, occupation, self-reported household economic status, region, political self-identification, perceived risk of COVID-19 infection, perceived safety of a COVID-19 vaccine, and trust in the government. Perceived risk of COVID-19 infection was assessed based on two aspects of risk perception: affective and cognitive [25,26]. The affective risk perception of COVID-19 was assessed using the question, “How worried are you about being infected with COVID-19?” The responses of “Very worried” and “Somewhat worried” were classified as “Worried”, and the responses of “Not so worried” and “Not worried at all” were classified as “Not worried”. Cognitive risk perception of COVID-19 was assessed using the question, “How likely do you think it is that you could be infected with COVID-19?” The responses of “Very likely” and “Somewhat likely” were classified as “Likely”, and the responses of “Less likely” and “Not likely at all” were classified as “Not likely”. Perceived safety of a COVID-19 vaccine was assessed using the question, “How worried are you about the side effects of COVID-19 vaccine?” The responses of “Very worried” and “Worried” were classified as “Not safe,” and the responses of “Not really worried” and “Not worried at all” were classified as “Safe.” Trust in the government’s COVID-related countermeasures was assessed by asking about the respondents’ perceptions of how the government’s COVID-19-related countermeasures had performed. Trust in government was measured by evaluating COVID-19 countermeasures based on the concept of competence-based trust [27,28]. The responses were “No opinion or neutral,” “Appropriate action,” and “Inappropriate action.” The details of the questionnaire items can be found in Supplementary File S2.

### 2.3. Analysis

We performed univariate analysis using the chi-square test to identify the relationship between participants’ hesitancy to receive a hypothetical COVID-19 vaccine and each demographic parameter. The missing values of the independent variables were ≤7.5% (cognitive risk perception of COVID-19, 7.5%; perceived performance of the government’s COVID-related countermeasures, 3.2%; perceived safety of vaccine, 2.6%; and the remainder included the self-reported household economic status, etc.). Missing values were omitted only when the statistics were calculated without being removed.

We then analyzed the factors affecting vaccine hesitancy using multivariate logistic regression. Our multivariate logistic regression model was adjusted for gender, age, occupation, self-reported household economic status, regions, political ideology, risk perception of COVID-19 (affective and cognitive), perceived safety of vaccine, and perceived performance of the government’s COVID-19-related countermeasures. For the logistic regression for hesitancy toward the hypothetical COVID-19 vaccine, “y = 1” was used for “Will not receive the vaccine” and “y = 0” was used for “Will receive the vaccine.” Of the dependent variables, 9.8% were missing (do not know/refuse).

### 2.4. Ethics Approval Statement

This study was reviewed and approved by the Institutional Review Board (IRB) of Seoul Metropolitan Government-Seoul National University Boramae Medical Center (IRB No. 07-2021-13). The need for informed consent was waived by the IRB due to the fact that the data were analyzed anonymously.

### 2.5. Patient and Public Involvement

There was no patient or public involvement in the design or planning of this study.

## 3. Results

### 3.1. Demographic Factors

Table 1 presents the general characteristics of the participants. Participants’ vaccine hesitancy statistically and significantly differed according to all demographic factors except for occupation, self-reported household economic status, region, and political belief We found that vaccine hesitancy was higher in women, younger participants, those who perceived lower levels of risk from COVID-19, those who doubted safety of a COVID-19 vaccine, and those who perceived the government’s COVID-19-related countermeasures as ineffective or inappropriate. 

### 3.2. Factors Associated with Vaccine Hesitancy

Table 2 illustrates the association between the independent variables and study participants’ vaccine hesitancy. The results show that age, political belief, affective risk perception of COVID-19, cognitive risk perception of COVID-19, perceived safety of a COVID-19 vaccine, and perceived performance of the government’s COVID-19-related countermeasures were all significantly correlated to vaccine hesitancy. Patients younger than 50 years of age were more hesitant about a hypothetical COVID-19 vaccine (adjusted OR [aOR] 2.03–2.20; 95% confidence interval [CI] 1.04–4.35). All political beliefs, except for “moderate,” were positively associated with vaccine hesitancy. Liberals (aOR 1.96; 95% CI 1.12–3.42) and participants with no political opinion (aOR 2.02; 95% CI 1.02–4.00) were more hesitant about receiving a potential vaccine than conservatives. Those who perceived the affective and cognitive risks of COVID-19 were more likely to receive such a vaccine. Those who perceived the vaccine as unsafe had great vaccine hesitancy (aOR 5.29; 95% CI 2.95–9.47), as were those who perceived the performance of the government’s COVID-19-related countermeasures as ineffective or inappropriate (aOR 3.45; 95% CI 2.25–5.27) or had a neutral stance on those measures (aOR 3.38; 95% CI 1.73–6.58).

## 4. Discussion

The aim of present study was to examine the associations among political orientation, trust in the government, and COVID-19 vaccine hesitancy. Quite possibly, this study is the first to explore evidence of the correlation between political orientation, government trust, and COVID-19 vaccine hesitancy during the COVID-19 pandemic in South Korea [11,13,16,17,18,19,20,21,25].

First, study participants who identified as liberal or “no political opinion” had higher rates of vaccine hesitancy than those who identified as conservative. These results differ from previous studies which have reported that conservativism and vaccine hesitancy are related [13,14,22,29]. It is possible that safety concerns surrounding the Oxford–AstraZeneca COVID-19 vaccine that surfaced in Europe in March 2021 influenced these individuals’ vaccine hesitancy [30]. Further research is needed to better understand the relationship between political beliefs and vaccine hesitancy in other contexts and why this relationship appears to be so different in the South Korean context. To deal most effectively with the phenomenon that vaccine hesitancy depends on individual political tendencies, our results show that it is necessary to classify political tendencies individually and strengthen vaccination promotion strategies.

Second, we found that the lower people’s trust in the government was, the higher their rate of vaccine hesitancy. This finding is corroborated by previous studies [20,31]. The lower the trust you have in the government, the more sensitive the risk conception to the threat, and the more sensitive it can be to the government for providing safe vaccines [25]. Government trust and social networks have been found to play important roles in determining people’s health behaviors [13,15,20,31,32]. Thus, they should be researched in more detail in the future. The Republic of Korea has undergone two general elections during the period of the Middle East respiratory syndrome (MERS) and COVID-19. During its spread in 2003, the Conservative Party was in power. The Progressive Party is currently in control of the COVID-19 situation. Despite the spread of COVID-19 in 2021, the electorate wore sanitary gloves at the time of the general election and expressed their expectations for the Progressive Party, with a turnout of over 66%. South Korea has long held differing political tendencies for each region, with the Progressive Party predominating Honam region and the Conservative Party predominating the Yeongnam region. However, as the COVID-19 situation continues, the presidential approval rating of the Progressive Party has recently been declining steadily, regardless of the region. Thus, South Koreans are sensitive to the current situation of COVID-19, and their perception of the public health crisis is affecting political habits. Various studies suggest that vaccine hesitancy tends to vary depending on political orientation and vaccine awareness [12,14,15,16]. Therefore, it is necessary to diversify communication channels by classifying those who recommend active vaccination and those who misdeliver vaccination. In addition, further political tendency research is necessary to understand the hidden meaning of vaccine hesitancy.

Third, we found that those who perceived their risk of contracting COVID-19 as lower or the risk of the vaccine having dangerous side effects as higher, the higher their tendency toward vaccine hesitancy. Vaccine hesitancy cannot lead to being vaccinated if one is concerned about the side effects caused by the vaccine [33]. Attitudes toward the vaccination can be determined depending on the balance between COVID-19 infection, the risk of vaccine’s side effects, and attitudes [34]. Thus, we suggest that information about COVID-19 and the safety of a COVID-19 vaccine should be provided to the public in a transparent method to improve vaccine inoculation rates. Transparency here means providing a variety of information about the vaccine’s effectiveness, side effects, etc., especially to people and groups who are sensitive to vaccine safety issues [35]. However, it is important for the government and related professionals to maintain trust by communicating effectively [20]. 

Fourth, we looked at whether the demographic characteristics were related to vaccine hesitancy and found higher vaccine hesitancy in younger (<50) people. This finding is similar to those of previous studies [35,36]. Further research is needed to identify the factors affecting the phenomenon of vaccine hesitation in young people. This could lead to higher vaccination rates among younger people who are less aware of the risks of COVID-19.

This study has a few limitations. First, since we utilized cross-sectional survey data, the relationship cannot be confirmed. Second, the results of this survey may not predict actual inoculation rates since it is a survey on hesitancy, which exists on a spectrum from action to non-action, and due to the fact that it measured people’s reactions to a hypothetical COVID-19 vaccine prior to the actual distribution of vaccines. Third, due to the opacity of the vaccine supply and order, the investigation was conducted in a method that did not include a separate validation of the vaccine hesitancy questions. Lastly, co-morbidities of participants were not included in our survey data.

## 5. Conclusions

This study aimed to reveal the associations among people’s political beliefs, government trust, and hesitancy to receive a hypothetical COVID-19 vaccine in South Korea. This study suggests that even if the safety of a vaccine and risk perception of COVID-19 are adjusted, self-rated political ideologies and government distrust can be associated with COVID-19 vaccine hesitancy. To resolve vaccine hesitancy, the government needs to put more effort into communication with those who are liberal or “no political opinion”, younger, and have low levels of government trust. To create a safe everyday environment against COVID-19 in the long-term, it is important to increase the vaccination rate. In addition to the 3Cs (confidence, complacency, and convenience) that affect vaccine hesitancy, policy development is required to consider additional political tendencies, government trust, etc. Further investigation is required to understand the mechanism of COVID-19 vaccine uptake for effective herd immunity.

## Figures and Tables

**Table 1:** Respondents’ general characteristics.

Variables	Total Respondents (%)	Hesitancy regarding Hypothetical COVID-19 Vaccine	
		Hesitancy (%)	Acceptance (%)
Total	902 (100.0)	187 (20.8)	715 (79.2)
Gender *			
Male	458 (50.8)	80 (17.5)	378 (82.5)
Female	444 (49.2)	107 (24.1)	337 (75.9)
Age (years) *			
18–29	162 (17.9)	49 (30.2)	113 (69.8)
30–39	136 (15.1)	36 (26.5)	100 (73.5)
40–49	174 (19.3)	39 (22.4)	135 (77.6)
50–59	178 (19.7)	30 (16.9)	148 (83.1)
≥60	253 (28.0)	35 (13.8)	218 (86.2)
Occupation			
Employed	559 (62.0)	115 (20.6)	444 (79.4)
Homemaker or student	220 (24.4)	49 (22.3)	171 (77.7)
Unemployed	123 (13.6)	24 (19.5)	99 (80.5)
Self-reported household economic status			
Upper	156 (17.6)	38 (24.4)	118 (75.6)
Middle	417 (47.2)	84 (20.1)	333 (79.9)
Lower	311 (35.2)	61 (19.6)	250 (80.4)
Region			
Yeongnam	229 (25.4)	51 (22.3)	178 (77.7)
Honam	90 (10.0)	15 (16.7)	75 (83.3)
Capital Metro	449 (49.8)	96 (21.4)	353 (78.6)
Chungcheong	93 (10.3)	17 (18.3)	76 (81.7)
Gangwon/Jeju	41 (4.5)	9 (22.0)	32 (78.0)
Political belief			
Conservative	225 (25.0)	37 (16.4)	188 (83.6)
Liberal	264 (29.3)	55 (20.8)	209 (79.2)
Moderate	274 (30.4)	61 (22.3)	213 (77.7)
No opinion	138 (15.3)	34 (24.6)	104 (75.4)
Affective risk perception of COVID-19 *			
Worried	656 (73.1)	112 (17.1)	544 (82.9)
Not worried	241 (26.9)	74 (30.7)	167 (69.3)
Cognitive risk perception of COVID-19			
Likely	566 (67.1)	109 (19.3)	457 (80.7)
Not likely	277 (32.9)	69 (24.9)	208 (75.1)
Perceived safety of vaccine *			
Not safe	635 (71.6)	165 (26.0)	470 (74.0)
Safe	252 (28.4)	19 (7.5)	233 (92.5)
Perceived performance of the government’s COVID-related countermeasures *			
Appropriate action	535 (61.1)	71 (13.3)	464 (86.7)
Inappropriate action	274 (31.3)	90 (32.8)	184 (67.2)
Neutral	67 (7.6)	20 (29.9)	47 (70.1)

* *p* < 0.05.

**Table 2:** Association between independent variables and vaccine hesitancy.

Variables	Vaccine Hesitancy
	Adjusted Odds Ratio (95% Confidence Interval)
Gender	
Male	0.74 (0.49–1.11)
Female	Reference
Age (years)	
18–29	2.11 (1.12–3.97) *
30–39	2.20 (1.11–4.35) *
40–49	2.03 (1.04–3.96) *
50–59	1.12 (0.57–2.18)
≥60	Reference
Occupation	
Employed	1.01 (0.52–1.97)
Homemaker or student	0.93 (0.45–1.92)
Unemployed	Reference
Self-reported household economic status	
Upper	1.00 (0.57–1.75)
Middle	1.04 (0.67–1.63)
Lower	Reference
Region	
Yeongnam	0.80 (0.32–2.01)
Honam	0.68 (0.24–1.98)
Capital Metro	0.75 (0.31–1.81)
Chungcheong	0.63 (0.22–1.80)
Gangwon/Jeju	Reference
Political belief	
Liberal	1.96 (1.12–3.42) *
Moderate	1.49 (0.88–2.52)
No opinion	2.02 (1.02–4.00) *
Conservation	Reference
Affective risk perception of COVID-19	
Worried	0.36 (0.24–0.56) *
Not worried	Reference
Cognitive risk perception of COVID-19	
Likely	0.64 (0.41–0.98) *
Not likely	Reference
Perceived safety of vaccine	
Not safe	5.29 (2.95–9.47) *
Safe	Reference
Perceived performance of the government’s COVID-related countermeasures	
Inappropriate action	3.45 (2.25–5.27) *
Neutral	3.38 (1.73–6.58) *
Appropriate action	Reference

* *p* < 0.05.

## Data Availability

No additional data are available.

## Supplementary Materials

The following are available online at https://www.mdpi.com/article/10.3390/ijerph182010655/s1, Supplementary file S1 Table S1: survey details, Supplementary file S2: Questionnaire.
