# Supplementary material for: Political Ideologies, Government Trust, and COVID-19 Vaccine Hesitancy in South Korea: A Cross-Sectional Survey
Source: Int J Environ Res Public Health. 2021 Oct 12;18(20):10655. doi: 10.3390/ijerph182010655 (PMC8536119; doi:10.3390/ijerph182010655)
Supplement: Supplementary file 1 [file ijerph-18-10655-s001.zip › 211009_Supplementary_Material_S1.pdf]

Number of elements in the Supplementary File: 1

Supplementary file S1

Table S1. Survey details

Period	Number of Eligible Cases	Number of Contacted Respondents	Number of Respondents Interviewed Successfully	CON2* (%)	COOP2* (%)	RR3* (%)
February 16–18, 2021	20,054	6,840	1,000	62.5	15.6	9.8

These surveys did not allow for a partial interview.
* Contact Rate 2 (CON2), Cooperation Rate 2 (COOP2), and Response Rate 3 (RR3) were calculated according to the American Association for Public Opinion Research (AAPOR) criterion.
