# Supplementary material for: Political Ideologies, Government Trust, and COVID-19 Vaccine Hesitancy in South Korea: A Cross-Sectional Survey
Source: Int J Environ Res Public Health. 2021 Oct 12;18(20):10655. doi: 10.3390/ijerph182010655 (PMC8536119; doi:10.3390/ijerph182010655)
Supplement: Supplementary file 1 [file ijerph-18-10655-s001.zip › 211009_Supplementary_Material_S2_questionnaire.pdf]

Number of elements in the Supplementary File: 1

Supplementary file S2: Questionnaire

Questionnaire

Vaccination hesitancy
If COVID-19 vaccines are introduced in Korea, will you receive or not receive the COVID-19 vaccine?

1. I will definitely receive the vaccine
2. I will probably receive the vaccine
3. I will probably not receive the vaccine
4. I will definitely not receive the vaccine
5. Don’t know/decline to answer

Perceived safety of vaccine
Are you worried that the COVID-19 vaccine will have side effects?

1. Very worried
2. Somewhat worried
3. Not really worried
4. Not worried at all
5. Don’t know/decline to answer

Political self-identification
To which of the following categories does your political tendency belong?

1. Very conservative
2. Conservative
3. Moderate
4. Liberal
5. Very liberal
6. Don’t know/decline to answer

Affective risk perception of COVID-19
A new coronavirus infection, COVID-19, has been spreading recently.
How worried are you about being infected with COVID-19?

1. Very worried
2. Somewhat worried
3. Not so worried
4. Not worried at all

Cognitive risk perception of COVID-19
How likely do you think it is that you could be infected with COVID-19?

1. Very likely
2. Somewhat likely
3. Less likely
4. Not likely at all

Perceived performance of government's countermeasures

Do you think the incumbent government is taking appropriate actions to address COVID-19?

1. Taking appropriate actions
2. Taking inappropriate actions
3. Neutral
4. Do not know

Gender

Please select your gender.

1. Male
2. Female

Age

Please specify your age.

Occupation

What is your occupation?

1. Farming/forestry/fishery
2. Self-employed
3. Blue collar (sales/services, functional/skilled worker, general worker)
4. White collar (office/technical position, management, professional/freelancer)
5. Homemaker
6. Student
7. Unemployed
8. Retired
9. Other (Specify: ______________)

Self-reported household economic status

If you divide the standards of living of Korean people into five levels—upper, upper middle, middle, lower middle, and lower—to which level do you think your household's living standard belongs?

1. Upper
2. Upper middle
3. Middle
4. Lower middle
5. Lower

Area

Which region do you live in? Please answer based on your address.

*We do not have your location information because respondents have been randomly selected and called via phone.

1. Seoul
2. Busan
3. Daegu
4. Incheon
5. Gwangju
6. Daejeon
7. Ulsan
8. Sejong

9. Gyeonggi
10. Gangwon
11. Chungbuk
12. Chungnam
13. Jeonbuk
14. Jeonnam
15. Gyeongbuk
16. Gyeongnam
17. Jeju
